# Learn from antibody–drug conjugates: consideration in the future construction of peptide-drug conjugates for cancer therapy

**DOI:** 10.1186/s40164-022-00347-1

**Published:** 2022-11-08

**Authors:** Mo Wu, Wei Huang, Nan Yang, Yanyong Liu

**Affiliations:** 1 Department of Pharmacology, Institute of Basic Medical Sciences, Chinese Academy of Medical Sciences & School of Basic Medicine, Peking Union Medical College, Beijing, 100005 China; 2 Medical College, Tibet University, Lhasa, 850000 Tibet Autonomous Region China

**Keywords:** Antibody–drug conjugates, Peptide-drug conjugates, Drug delivery, Cancer therapy

## Abstract

Cancer is one of the leading causes of death worldwide due to high heterogeneity. Although chemotherapy remains the mainstay of cancer therapy, non-selective toxicity and drug resistance of mono-chemotherapy incur broad criticisms. Subsequently, various combination strategies have been developed to improve clinical efficacy, also known as cocktail therapy. However, conventional “cocktail administration” is just passable, due to the potential toxicities to normal tissues and unsatisfactory synergistic effects, especially for the combined drugs with different pharmacokinetic properties. The drug conjugates through coupling the conventional chemotherapeutics to a carrier (such as antibody and peptide) provide an alternative strategy to improve therapeutic efficacy and simultaneously reduce the unspecific toxicities, by virtue of the advantages of highly specific targeting ability and potent killing effect. Although 14 antibody–drug conjugates (ADCs) have been approved worldwide and more are being investigated in clinical trials so far, several limitations have been disclosed during clinical application. Compared with ADCs, peptide-drug conjugates (PDCs) possess several advantages, including easy industrial synthesis, low cost, high tissue penetration and fast clearance. So far, only a handful of PDCs have been approved, highlighting tremendous development potential. Herein, we discuss the progress and pitfalls in the development of ADCs and underline what can learn from ADCs for the better construction of PDCs in the future.

## Introduction

At present, cancer is the second cause of death worldwide only behind cardiovascular diseases. In 2020, cancer contributed to 10 million deaths and 19.3 million new diagnosed cases globally and the cancer burden is estimated to rise to 28.4 million new cancer cases in 2040, urging the need to develop novel therapeutic approaches to improve clinical efficacy [1].

The introduction of sulfur mustard opened the prelude to the modern era of cancer chemotherapy [2]. Since then, a large number of chemotherapeutic drugs have been discovered or synthesized to combat different cancers [3]. Clinically, cytotoxic agents are systemically administered to eradicate the rapidly dividing cancer cells, but unfortunately affecting healthy proliferative cells. Non-selective toxicity and subsequent drug resistance of mono-chemotherapy attract the most common criticism. In recent years, understanding of the molecular, cellular and systemic processes driving cancer initiation, progression, heterogeneity and metastatic spread has evolved tremendously and a plethora of precision medicines spring up [4]. However, high tumor heterogeneity seriously challenges mono-chemotherapy, reflected by frequent drug resistance and tumor recurrence [5–7].

Therefore, numerous efforts have been made to explore the combination regimens, where multiple agents are co-administrated to simultaneously modulate multiple signaling pathways and offer significant benefits [8, 9]. Although remarkable efficacy has been witnessed with combination chemotherapy, clinical outcomes of conventional “cocktail administration” are not always as good as anticipated. For example, it is difficult to control the desired levels in the tumor tissues for the combined drugs with different pharmacokinetic properties, which is vital to the ultimate clinical outcomes [10]. To cope with those challenges, an integrated strategy is introduced to reduce the serious side effects and elevate the drug concentration where needed, which stresses a shift from the cocktails to conjugate combination [11].

**Fig. 1:**
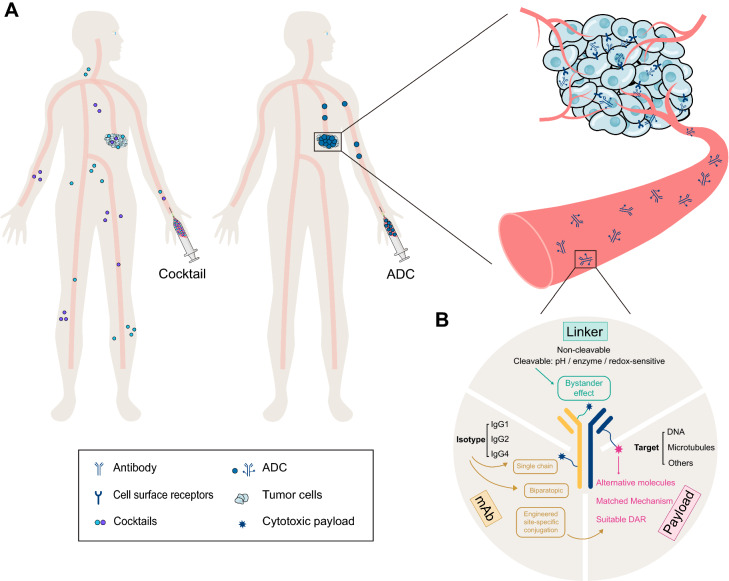
Antibody–drug conjugate strategy significantly improves the antitumoral efficacy. **A** ADCs exhibit superior tumor targeting in the circulation compared with cocktail therapy. **B** Three main components of an ADC include antibody, linker, and payload

Antibody–drug conjugates (ADCs), which are developed based on the concept of “magic bullet” conceived by Paul Ehrlich 100 years ago, enable the targeting delivery of toxic drugs to cancer cells, providing an opportunity for delivering two distinctive therapeutic entities, the antibody and the small molecule, in an integrated pattern [12] (Fig. 1A). ADCs have been widely used for cancer treatments, exhibiting both high efficacy and favorable tolerability. By the end of December 2021, twelve ADCs have been approved by the FDA (Table 1), and it is estimated that the global sales of ADCs will exceed $16.4 billion by 2026 [13]. However, high cost, as well as low solid tumor penetration due to large molecular size and emerging resistance, undermine the development of ADCs [14]. Peptide-drug conjugates (PDCs) and ADCs share similar concepts, but with differential structures and properties. PDCs use a peptide as a carrier and offer some unparalleled benefits, including enhanced tumor penetration, reduced immunogenicity, and lower production costs [15, 16].

**Table 1:** ADCs currently approved by the US FDA before December 2021

ADC	Target antigen	mAb isotype	Linker	Payload
Gemtuzumab ozogamicin (Mylotarg)	CD33	IgG4	Cleavable (Hydrazone)	Ozogamicin
Brentuximab Vedotin (Adcetris)	CD30	IgG1	Cleavable (Peptide)	MMAE
Ado-trastuzumab emtansine (T-DM1) (Kadcyla)	HER2	IgG1	Non-cleavable (Thioether)	DM1
Inotuzumab Ozogamicin (Besponsa)	CD22	IgG4	Cleavable (Hydrazone)	Ozogamicin
Moxetumomab pasudotox-tdfk (Lumoxiti)	CD22	IgG1	Cleavable (Peptide)	PE38
Polatuzumab vedotin-piiq (Polivy)	CD79b	IgG1	Cleavable (Peptide)	MMAE
Enfortumab vedotin-ejfv (Padcev)	Nectin-4	IgG1	Cleavable (Peptide)	MMAE
Fam-trastuzumab deruxtecan-nxki (T-DXd) (Enhertu)	HER2	IgG1	Cleavable (Peptide)	DXd
Sacituzumab govitecan-hziy (Trodelvy)	TROP2	IgG1	Cleavable (Peptide)	SN-38
Belantamab mafodotin-blmf (Blenrep)	BCMA	IgG1	Non-cleavable (Thioether)	MMAF
Loncastuximab tesirine-lpyl (Zynlonta)	CD19	IgG1	Cleavable (Peptide)	SG3199
Tisotumab vedotin-tftv (Tivdak)	Tissue factor	IgG1	Cleavable (Peptide)	MMAE

Here, we briefly reviewed the advances and challenges in the development of ADCs. Then we focus on what we can learn from ADCs to provide potential insights for future efforts to overcome these roadblocks and better design of PDCs.

## Challenges in the ADCs development

Key components for ADCs construction comprise the selected target, antibody, cytotoxic payload and a covalent linker (Fig. 1B). The majority of ADCs share a similar pattern of action: upon binding to the membrane target, the ADC is internalized and trafficked to the lysosome, where the covalent linkage is cleaved to release the payload [17, 18]. The released payload subsequently interacts with its intracellular target to exert cytotoxicity. Meanwhile it may diffuse and kill the neighboring cells to induce a so-called “bystander effect” [19].

**Fig. 2:**
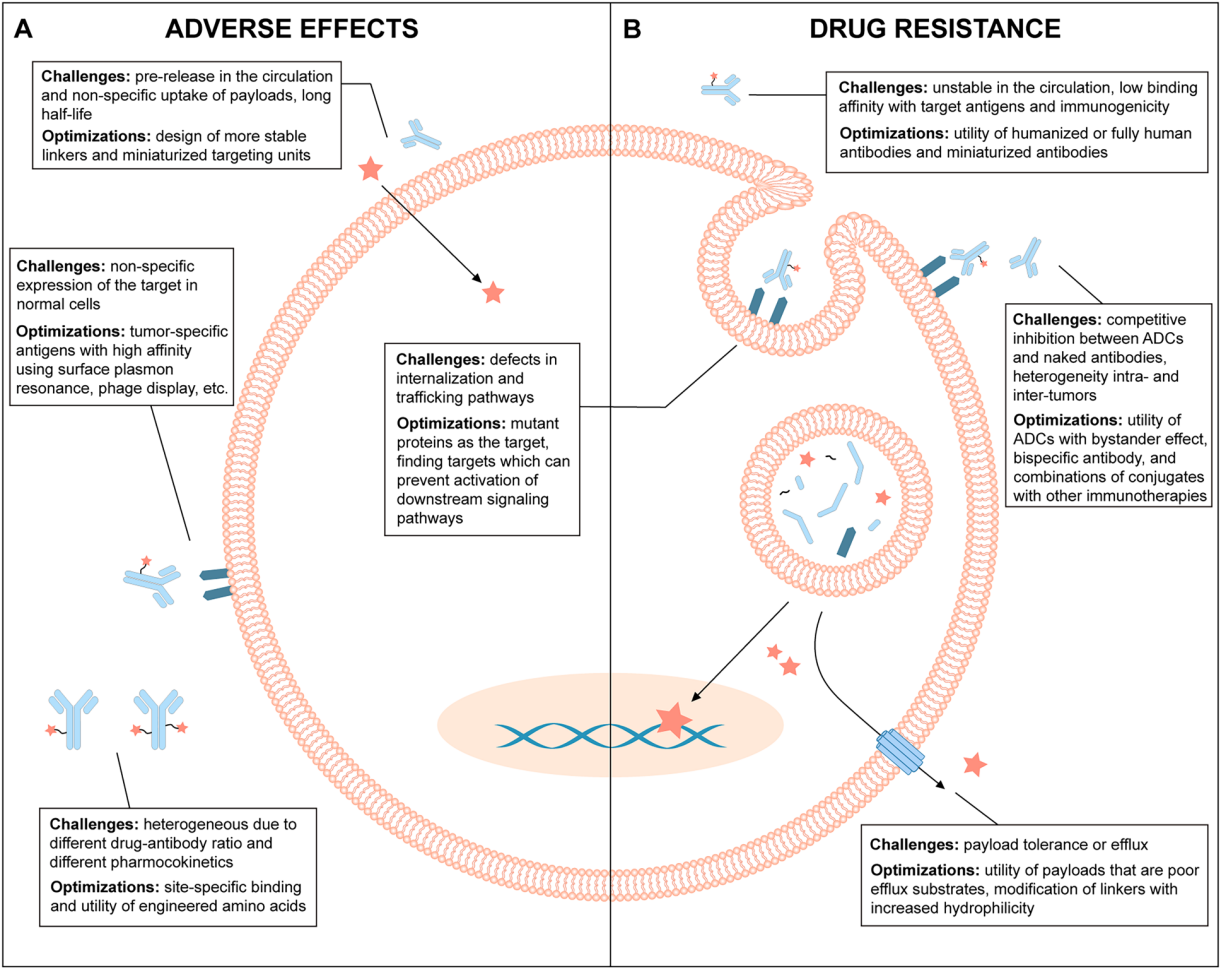
Overview of the current challenges and future optimizations of ADCs. **A** Mechanism underlying the adverse effects of ADCs and optimizing strategies. **B** Resistant mechanisms of ADC and optimizing strategies

ADC is initially conceived to increase their specific retention and cytotoxic activity at the tumor sites while sparing the healthy tissues. An ideal ADC should remain stable in the circulation and accurately target cancer cells. Each element can affect the final efficacy and safety of an ADC and insufficient consideration will set up obstacles for future clinical translation. Despite the growing interest, challenges remain to expand their therapeutic index for the development of ADCs (Fig. 2).

### Undesired toxicity

#### On-target related toxicity

The successful development of an ADC seriously relies upon an appropriate target antigen for antibody binding. Ideally, to reduce off-target toxicity and provide an acceptable therapeutic index for an ADC, target antigens should be highly expressed in tumors, but low or even not in normal tissues, or at least limited to a given tissue type [20, 21]. Unfortunately, tumor cells usually display tumor-associated antigens (TAA) instead of completely specific antigens (tumor-specific antigens, TSA) [22]. Unless the antigen-expressing normal cells are insensitive to drug action, the undesired uptake of an ADC will lead to toxicity and decrease ADC doses available to the tumor [23].

Antibody conjugates of BR96-DOX were prepared by coupling the chimeric monoclonal antibody BR96 to the anticancer drug doxorubicin (DOX). The monoclonal antibody binds an antigen related to Lewis^Y^ which is abundantly expressed at the surface of cells from many human carcinomas. Development of BR96-DOX was impeded by hemorrhagic gastritis, which was attributed to the unrecognized expression of Lewis^Y^ antigen on gastric mucosa cells [24, 25]. Similarly, bivatuzumab mertansine was discontinued due to the occurrence of skin toxicity in Phase I clinical trials in patients with advanced carcinoma. In addition to its expression on various carcinomas, including squamous cell carcinomas and a proportion of adenocarcinomas, CD44v6 is also expressed on normal proliferating epidermal skin cells [26]. HER2 is the antigen of the two recently approved antibody conjugates of Enhertu (T-DXd) and Kadcyla (T-DM1). It is upregulated in multiple types of tumors including breast cancer. However, it is also expressed in many healthy tissues, such as respiratory, gastrointestinal and reproductive tissues, potentially mitigating this advantage. When Kadcyla was used for the treatment of HER2-positive breast cancer, the side effects of nodular regenerative hyperplasia and corneal abnormalities were observed [27, 28]. Side effects in patients treated with the EGFR-targeted ADC (depatuxizumab mafodotin) for recurrent glioblastoma included corneal abnormalities [29]. A folate receptor α-targeted ADC (mirvetuximab soravtansine) also showed corneal abnormalities following treatment of platinum-resistant ovarian cancer patients [30]. Treating non-Hodgkin's lymphoma patients with CD79b-targeted ADC (polatuzumab vedotin) resulted in peripheral neuropathy in 70% of patients [31].

Identification and validation of adequate antigenic targets for the antibody component still remain an arduous obstacle in the clinical success of ADCs. Based on advances in sequencing, machine learning, and information sharing, the successful development of robust cancer neoantigen prediction strategies likely have a significant impact, with the potential to facilitate superior ADC design.

#### Linker instability-associated toxicity

The choice of suitable linkers is likewise the challenge for the development of effective ADCs. As a small and central part of ADCs, a linker is designed to be stable in the bloodstream and subsequently release the drug in its active form within or close to the target cells [32, 33]. The role of linker is fundamental for efficient delivery of the cytotoxic drug, but it is also the determinant factor to the toxicity of an ADC product. Premature release of drugs in the circulation can result in systemic toxicity and a lower therapeutic index.

Neutropenia is a kind of common toxicity for many ADCs conjugated to MMAE via protease cleavable valine-citrulline linkers such as Brentuximab vedotin, ASG-5ME, Glembatumumab vedotin, Indusatumab vedotin, Polatuzumab vedotin and PSMA-targeting ADC [20, 34–38]. Results showed that vc-MMAE-based ADC-induced neutropenia is due to a direct cytotoxic effect of released payload on differentiated neutrophils in the bone marrow [39]. Peripheral neuropathy is another important target-independent clinical toxicity associated with microtubule inhibitor ADCs leading to treatment discontinuation and/or dose reduction [40]. Microtubule inhibitors disrupt interphase microtubule function critical for the active transport of key essential proteins from the neuron cell body to distal synapses and ultimately result in peripheral neuropathy [41].

The mechanism of drug release is an important consideration in linker selection. Both cleavable and non-cleavable linkers have been used in approved second‐generation ADCs and in third-generation ADCs that are currently being investigated in clinical trials [42, 43]. Collectively, linker stability during several days in the circulation and efficient cleavage upon delivery into the target cell, should be considered for effective linker design.

#### Bystander-effect associated toxicity

After uptaken by target antigen-positive cells, released payload from ADCs may also be cytotoxic to adjacent target antigen-negative cells called the bystander effect [44]. The free payload can either passively enter the extracellular space or be released due to loss of membrane integrity, namely passive diffusion, transporter-mediated uptake, or other non-specific endocytosis mechanisms to cause cytotoxicity [19]. The bystander effect in ADCs is often associated with increased tumor killing (efficacy), especially for tumors with heterogeneous antigen expression [45]. For example, the cleavable ADC DS-8201a (Enhertu) releases its membrane permeable payload DXd, which kills HER2-positive cells surrounding the targeted cancer cells, but not more distant cells. This is beneficial for the treatment of HER2 heterogeneous tumors [46]. Recently, a phase 3, multicenter, open-label, randomized trial to compare the efficacy and safety of trastuzumab deruxtecan (a HER2 antibody–drug conjugate) with those of trastuzumab emtansine in patients with HER2-positive metastatic breast cancer previously treated with trastuzumab and taxane was conducted. Results showed that the risk of disease progression or death was lower among those who received trastuzumab deruxtecan than those who received trastuzumab emtansine which employs a stable linker without bystander effects [47]. The bystander effect is advantageous for tackling heterogeneous tumors and penetrating deeper into solid tumors which are less accessible to the conjugate.

However, cleavable linkers do not always enable the bystander effect, rather it depends on membrane-penetrability and charge properties of the released payload [48]. Moreover, the increased cellular permeability needed to achieve the bystander effect can also contribute to off-target toxicity. Released payloads permeate into normal tissues and lead to increased toxicity compared to non-cleavable, impermeable payload [49]. Hepatic toxicity was observed with Cantuzumab mertansine, targeting CanAg (tumor-associated carbohydrate antigen, a novel glycoform of MUC1) with a relatively labile SPP (N-succinimidyl 4-(2-pyridyldithio) pentanoate) linker. This effect is suggested to be due to bystander effect on adjacent normal hepatocytes [50].

Recent advances in ADC technology have brought about cytotoxic payloads able to be metabolized in cancer cells to membrane impermeable metabolites. This approach may control the bystander effect, retaining beneficial chemical properties for killing cancer cells while also significantly minimizing systemic toxicity to normal cells.

#### Receptor-mediated toxicity

Target-independent uptake and toxicity of ADCs also are mediated by different candidate receptors which recognize the Fc (fragment crystallizable) region in the IgG backbone in ADCs. IgG constant domains are highly conserved in the structure allowing interaction with other components of the immune system through Fc receptors to initiate effector immune functions. Although Fc-mediated effector functions are not typically required for achieving ADC efficacy, recognition and binding of Fc receptors to the antibody (IgG) component of ADCs could mediate target-independent internalization to normal cells [51]. Other Fc binding receptors including Fc gamma receptors (FcγRs), neonatal Fc receptor (FcRn) and C-type lectin receptors (CLRs) may potentially mediate IgG/ADC internalization/trafficking and toxicity to normal cells [37, 52, 53].

### Drug resistance

With high plasticity, tumors can develop resistant mechanisms to overcome the hostile challenges and thereby limit the success of the treatment. As for ADCs, tumor can adopt multiple alternations against individual components of the ADC. One mechanism of resistance involves the modulations in antigen recognition by the antibody through downregulation of the target from the cell surface, hindering the ADC from exerting their cytotoxic effect [54]. Several breast cancer cell lines were made resistant to T-DM1 by multiple cycles of exposure to an anti-HER2 trastuzumab-maytansinoid ADC structurally similar to T-DM1. The resistant cells exhibited a markable decrease in HER2 protein levels after several months from the initiation of the treatment [55, 56].

Another common mechanism of drug resistance is the removal of the payload via ATP-binding cassette transporters [57]. As the potential substrates for these pumps, the cytotoxic warheads used in ADCs may be expelled out of the target cell so as to reduce the drug efficacy. Clinical data revealed that efflux pumps are attributed to the reduced efficacy of gemtuzumab ozogamicin [58]. Elevated drug transporter protein expression has also been observed in T-DM1 resistant cells in addition to decreases in surface antigen expression [59].

Activation of downstream signaling pathways likewise contribute to the acquisition of resistance to ADCs, together with other potential mechanisms of resistance to ADCs could be mutations in the cellular target for the cytotoxic agent, defects in internalization, trafficking, and recycling, lysosomal degradation leading to impairment of drug release, and alterations in cell death pathways [54].

Altogether, resistance to ADCs has been one of the critical factors that have limited the clinical success of these drugs. The optimization in the structure of ADCs may be helpful to develop new compounds capable of overcoming resistance.

## Overview of progress and advantages of PDCs vs. ADCs

**Fig. 3:**
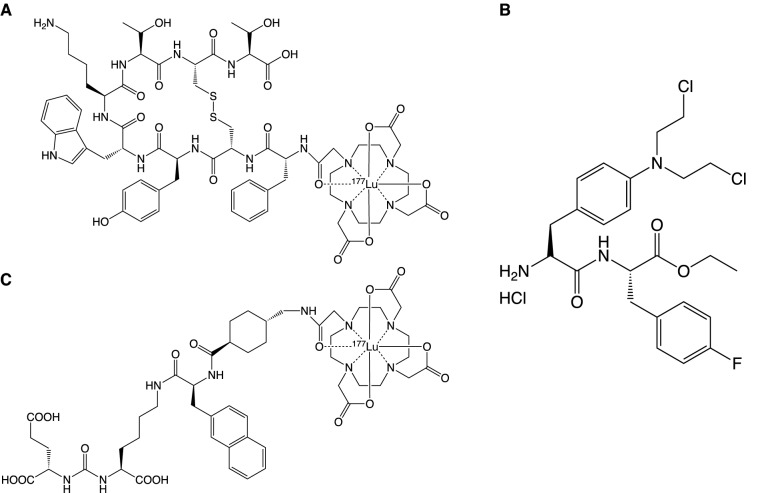
Structures of three FDA approved PDCs. **A** Lutathera. **B** Pepaxto. **C** Pluvicto

PDCs share a similar concept with ADCs, but with differential structures and properties. Generally speaking, the high penetration into solid tumors and low production cost make the PDC an attractive alternative [11]. Several radioactive PDCs have been approved for diagnostic imaging previously, such as ^111^In-DTPA-octreotide and ^68^ Ga-DOTATATE [60]. Until now, three therapeutic PDCs, Lutathera (Fig. 3A), Pepaxto (Fig. 3B) and Pluvicto (Fig. 3C), have been approved by the FDA. Lutathera consists of a somatostatin agonist (octreotate), a chelating molecule (DOTA), and a beta-emitting radioisotope (^177^Lu), which is the first therapeutic PDC developed by Novartis [61]. Pepaxto is intended for multiple myeloma and other hematologic malignancies, immunoglobulin light chain amyloidosis, and solid tumors. It is constructed by linking melphalan, a DNA alkylating agent, with the peptide targeting aminopeptidase, which can release melphalan rapidly in tumor cells [62]. However, the results of the phase III randomized controlled trial led to the withdrawal from the U.S. market in October 2021 due to the poor overall survival rate in the treatment of multiple myeloma [63]. Pluvicto belongs to radioligand therapeutic agent, constructed by coupling a PSMA-binding ligand to a DOTA chelator radiolabeled with lutetium-177 [64]. Although Pluvicto is classified as a radionuclide-drug conjugate (RDC), its drug structure is similar to that of Lutathera. Pluvicto is developed for the treatment of PSMA-positive mCRPC [65]. Several PDCs are ongoing in clinical trials, indicating the huge potential and market prospect of PDCs [60]. The employed linkers, peptides and targets are shown in Table 2. And PDCs under phase II or phase III development are shown in Table 3.

**Table 2:** Linkers, peptides and targets of some PDCs [139, 159, 160]

Linker			Peptide	
Type	Name	Structure	Name	Target
Non-cleavable	Thioether bond		Octreotide	SST2
	Oxime bond		Octreotate	SST2
	Triazole bond		NGR	CD13
pH sensitive	Hydrazone bond		Angiopep-2	LRP-1
	Acetal/Ketal bond		GnRH	GnRH-R
Enzyme sensitive	Ester bond		Lys6-modified GnRH	GnRH-R
	Amide bond		RGD	αv integrins
	Carbamate bond		c(RGDfK)	αv integrins
	Val-Cit		iRGD	αv integrins
	Ala-Ala-Asn		Bombesin(7–14)	Bombesin receptors
	Gly-Phe-Leu-Gly		TH19P01	Sortilin receptor
Redox sensitive	Ddisulfide bond		Bicycle	MMP-14

**Table 3:** PDCs under phase III or phase II development

Name	Phase	Target antigen	Payload	Refs
GRN1005	3	LRP-1	Paclitaxel	[161]
AEZS-108	3	LHRH receptor	DOX	[162]
EP-100	2	LHRH receptor	Cationic lytic peptides	[163]
BT1718	1/2	MT1-MMP	Mertansine (DM1)	[159]
BT5528	1/2	EphA2	MMAE	[123]
BT8009	1/2	Nectin-4	MMAE	[124]
CBX-12	1/2	Topoisomerase I (TOP1)	Exatecan (DX-8951f)	[164]
L-377202	1/2	Prostate-specific antigen (PSA)	DOX	[165]
PEN-221	1/2	Somatostatin receptor 2 (SSTR2)	Mertansine(DM1)	[166]

### Low molecular weight than mAbs

Compared with antibodies, peptides used as a carrier to target tumor cells are usually less than 40 amino acid residues [16], which offers some unparalleled benefits. The small molecular weight makes the peptide less immunogenic than antibodies. Furthermore, the production cost is lower, and the drug load is easier to control, which facilitates to produce homogeneous conjugates [16, 66]. At the same time, peptides are easily modified to improve their physiological stability and pharmacokinetics and narrow the gap between PDCs and ADCs.

Peptides used in PDCs fall into two categories: cell-penetrating peptides (CPPs) and tumor-homing peptides (THPs). PDCs with CPPs enter cells by non-specific mechanisms, while PDCs with THPs mediate cytotoxic payloads into tumor cells selectively by specifically binding to their targets or receptors expressed on the tumor cell surface. Due to the low cellular specificity and low circulating half-life of CPPs [67], the application of these PDCs is limited. In contrast, THPs show similar effectiveness as mAbs and are widely used due to their low production costs and immunogenicity.

CPPs are up to 40 amino acids long peptides, with the ability to penetrate cell membranes by various mechanisms [67]. Several studies showed that linking drugs to CPPs can increase their efficiency by promoting intracellular delivery [68–70]. However, so far, there are none of the CPP-binding drugs approved by FDA, and some clinical trials have been terminated, possibly due to poor circulation stability, lack of specificity and selectivity, the low release efficiency of drugs, and systemic toxicity [71]. Therefore, how to maximize the targeting efficiency is crucial for the development of PDCs using CPPs. There are a few methods used to improve the ability of CPPs to target tumor cells, such as building specificity into tumor-targeting CPP constructs (also called tumor-homing CPPs) using phage display technologies. Zhou et al. found a novel CPP-MT23 with mouse melanoma cell specificity, which can only enter B16 melanoma cancer cells without any cytotoxicity, based on phage display and an in silico approach [72]. Other optimization methods include designing and developing CPP-drug conjugates coupled with THPs to target specific tumor markers, or activating CPPs in the tumor microenvironment to enhance affinity to tumors, so that conjugates can be specifically internalized and release payloads after reaching the tumor site.

THPs are a kind of peptide selected to specifically target proteins expressed on tumors, which can realize targeted delivery based on the principle of receptor/ligand targeting [73]. One peptide used as a THP is the RGD peptide (Arg-Gly-Asp), which widely exists in extracellular matrix proteins and can be specifically recognized by integrins. RGD peptide specifically binds with a variety of integrins, activates conduction pathways, and then promotes a series of physiological behaviors such as adhesion, migration, infiltration, and proliferation [74]. Studies have shown that integrins are associated with a variety of cancers, such as colorectal cancer, melanoma, prostate cancer, breast cancer, glioblastoma, lung cancer, and thyroid cancer [75]. Therefore, several integrin-targeting agents have been designed, especially for αvβ3 receptors, some of which have entered clinical trials. For details, please refer to the review of Li et al. [76]. PDCs with the RGD peptide targeting integrin are detailed in a review by Chatzisideri et al. and summarized in Table 1 [77]. Almost all integrins can bind to the extracellular matrix (ECM) via RGD motifs, which are used as the carrier for integrin-targeted drug delivery systems, and most of them are based on RGD motifs or modified RGD motifs, such as cRGDfK [78] and cilengitide [79].

Gonadotropin-releasing hormone (GnRH), also known as luteinizing hormone-releasing hormone (LHRH), is a natural hormone whose receptor is overexpressed in a variety of cancers, including prostate cancers [80], endometrial cancers [81], and ovarian cancers [82]. Although GnRH receptor (GnRH-R) is expressed in the healthy tissues of reproductive organs and the pituitary gland, the GnRH-targeted drug delivery system (DDS) minimizes the adverse effect on the normal tissue by the virtue of physiological barriers [83]. Therefore, GnRH and its derivatives can be covalently linked to cytotoxic payloads for the GnRH-R-expressing cancer cells. For instance, AN-152 and AN-207, linked with DOX, successfully target GnRH-R positive cancer cell lines. However, AN-152 also exhibited cytotoxic activity against GnRH-R-negative cell lines. This might be attributed to the release of free DOX from the PDC in the blood by enzymatic cleavage or hydrolysis. Though proved to be effective and of low toxicity in women with GnRH-R-positive endometrial cancer in phase II trials, AN-152 did not improve overall survival, progression-free survival, overall response rate, clinical benefit rate, or adverse events compared to free DOX for advanced endometrial cancers in phase III clinical trials [83, 84]. Obayemi et al. conjugated D-Lys^6^-modified GnRH with prodigiosin (PGS) and paclitaxel (PTX) respectively for the treatment of triple-negative breast cancer (TNBC) cells in in vitro studies and both PDCs exhibited stronger antitumor activities than free PTX and in TNBC models [85].

### More choices of payloads than ADCs

Compared with ADCs, due to the good permeability and low molecular weight of peptides, the IC_50_ of payloads used in PDCs can be higher. The commonly used cytotoxins are divided into two categories. One is radionuclides, such as ^177^Lu, ^111^In, and ^90^Y. The other is cytotoxic drugs, such as gemcitabine (GEM), DOX, PTX and camptothecin (CPT). Boron neutron capture therapy (BNCT) is a targeting technology that has emerged in the past decade [86] and will not be included in this review.

Radionuclides can be used for cancer diagnosis and treatment in somatostatin analogs and peptide receptor radionuclide therapy (PRRT). Slightly different from the most typical structures of PDCs, radiolabeled somatostatin analogs typically contain three main components: a cyclic octapeptide (such as octreotide), a chelator (such as DTPA or DOTA), and a radioactive element (such as ^111^In, ^90^Y or ^177^Lu) [87]. The most commonly used bifunctional chelating agents are diethylenetriaminepentaacetic acid (DTPA) and 1,4,7,10-tetraazacyclododecane-1,4,7,10-tetraacetic acid (DOTA) [86]. [^111^In-DTPA]-octreotide (octreoscan) was the first available diagnostic radiolabeled somatostatin analog. However, studies have shown that ^111^In-coupled peptides are not efficient for PRRT, as the short distance traveled by Auger electrons after emission means that decay of ^111^In must occur close to the cell nucleus to be tumoricidal. Therefore, octreoscan is only approved for diagnostic imaging of somatostatin receptor (SSTR)-positive tumors. Replacing phenylalanine with tyrosine as the third amino acid in the octapeptide results in increased affinity to SSTR2, leading to the development of next-generation therapies of ^90^Y-DOTA, Try^3^-octreotide. This compound has DOTA instead of DPTA as the chelator, which allows stable binding of ^90^Y. The third generation SSTR targeted radionuclide therapy comprises ^177^Lu-DOTA, Tyr^3^-octreotate. The only difference between DOTA, Tyr^3^-octreotate and DOTA, Tyr^3^-octreotide is that the C-terminal threoninol of DOTA, Tyr^3^-octreotide is replaced with the threonine, improving binding to SSTR-positive tissues when compared with DOTA, Tyr^3^-octreotide [86]. Compared with ^90^Y-labeled counterparts, ^177^Lu-octreotate was very successful in terms of tumor regression, a survival benefit of several years, and improved quality of life. ^177^Lu is not a pure β emitter, but also emits low-energy γ rays, which allows direct posttherapy imaging and dosimetry [88].

Cytotoxic drugs used in PDCs can be classified according to their general mechanisms of action, including drugs that interfere with DNA replication and transcription (such as CPT and DOX), drugs that inhibit DNA biosynthesis (such as GEM and MTX), and anti-mitotic drugs acting on microtubules (such as PTX). CPT, a small molecule chemical drug, and its derivatives have been proved to possess potent antitumor and antiangiogenic activities, but their clinical applications are limited due to poor solubility and severe toxic side effects. Redko et al. found that coupling CPT to ALOS4 made CPT more stable than free CPT, accumulated in the nucleus, and induced intracellular damage [89]. PTX is a small-molecule cytotoxin targeting tubulin, which can inhibit cell division. However, it is highly hydrophobic and has P-glycoprotein-mediated efflux, leading to drug resistance. The conjugate 2PTX-OCT, with two PTX molecules, displayed a significant growth suppression of A549 tumors and had reduced toxicity [90]. Treatment with LHRH-conjugated PGS/PTX resulted in higher levels of necrosis in the tumors when compared to those treated with the unconjugated PGS or PTX drugs [85].

In addition, PDCs have higher and more controllable drug loading capacity compared with ADCs. Loading more payloads to a carrier can effectively improve drug concentration in target tissues. Lin et al. reported a rationally designed PTX drug amphiphile into well-defined supramolecular filaments that possess a fixed 41% paclitaxel loading, exerting effective cytotoxicity against several cell lines compared to that of free PTX [91].

## Learn from ADCs for future PDCs construction

With continued interests and commercial investment, more ADC candidates are currently under active clinical investigation as monotherapy or combinational therapy for various tumor types. We should be soberly aware that the development of ADC still faces major challenges such as undesired toxicity and drug resistance. In view that only a few PDCs have been approved, recent advances in antibody, payload, and linker optimization are worth learning to circumvent the pitfalls encountered with ADCs.

### Enhancing the tumor targeted killing effect while reducing the off-tumor toxicity

#### Exploration of novel target

To reduce off-target toxicity, an ideal target for both ADC and PDC should be expressed exclusively or predominantly in tumor cells, but rarely or low in normal tissues. As there is a high target overlap between two conjugate modalities, PDCs will inevitably encounter similar "on-target, off-tumor" toxicity to healthy tissue as ADCs do, highlighting the importance to explore novel tumor-specific antigens.

Recently, progress in gene sequencing technology and innovations in antigen discovery approaches have facilitated the identification of neoantigens. Some promising antigens have been identified including CD138 which is expressed in multiple myeloma and a variety of solid tumors, Trop 2 expressed on the cell surface of most solid tumors and mesothelin expressed in pancreatic and ovarian cancers [20, 92–96].

Besides membrane-located targets, stromal cell-targeted therapy gradually attracts researchers’ attention. The stromal cells in different types of tumors share some identical markers, therefore a drug conjugate targeting stromal cells could potentially be applicable for multiple tumor types. Several ADCs have been constructed based on targeting stromal cell antigens such as fibronectin [97], tenascin-C [98] and TEM8 [99]. Meanwhile, several peptides targeting the tumor extracellular matrix have been identified recently. Fibronectin is an important glycoprotein component of the extracellular matrix and is overexpressed in many malignant tumors. The fibronectin-fibrin complex is a significant biomarker for diagnosing cancers. A liner pentapeptide CREKA has been demonstrated to bind to the fibronectin-fibrin complex with good solubility, biocompatibility and targeting specificity [100–103]. Cathepsins are a family of endopeptidases expressed on the cell surface and then released to the extracellular matrix. They are overexpressed in various tumors, including breast, lung, colon, liver, gastric, ovarian and prostate cancers. The 4-mer peptide GFLG can be specifically hydrolyzed by cathepsin B to trigger drug release from drug carriers for effective tumor therapy [104–106].

The past few decades witnessed a fast development of artificial intelligence biology analysis algorithms, which can be catalogized as network-based biology analysis algorithms and machine learning-based (ML-based) biology analysis algorithms [107–110]. Network-based biology analysis algorithms provide a variety of alternative network approaches to identify cancer targets. ML-based biology analysis can not only efficiently handle high throughput, heterogeneous, and complex molecular data but also excavate the feature or relationship in the biological networks. By integrating gene expression profiles into genome-scale molecular networks, several therapeutic targets for cervical cancer have been identified, including receptors, microRNAs (miRNAs), transcription factors (TFs), proteins, and metabolites [111]. Laura et al. applied a consensus clustering algorithm that divided the network into sub-modules with different functions and demonstrated that F11R, HDGF, PRCC, ATF3, BTG2, and CD46 could be oncogenes and promising markers for pancreatic cancer [112, 113].

Moreover, under the support of functional genomics, Beha et al. performed genome-scale CRISPR–Cas9 screens in 324 human cancer cell lines from 30 cancer types and developed a data-driven framework to prioritize candidates for cancer therapeutics. They verified the Werner syndrome ATP-dependent helicase, as a synthetic lethal target in tumors from multiple cancer types with microsatellite instability [114].

Overall, we fully believe that the progress in neoantigen identification will greatly boost the development and clinical translation of drug conjugates with high efficiency and low toxicity.

#### Optimization of targeting units

Targeting moiety is an equally crucial component for a successful drug conjugate. With regard to ADCs, smaller binding units, such as peptide fragments, single-chain variable fragments, single-domain antibody fragments, or diabodies, have been employed to overcome the drawback of low penetration due to large size [115]. However, the utility of small targeting units may experience rapid clearance from circulation [116]. Therefore, several techniques have been developed to extend the half-life. For example, it is feasible to limit the degradation and elimination of peptides by modifying possible molecular chains to form probe (SIP)-tail, lactam bridges and stapling or clipping of peptide sequences or by cyclization [117]. Cyclization can prohibit or reduce the degradation, and in some cases, binding affinity of the peptide can be enhanced for improved stability [118]. After people discovered the RGD motif as the vascular-targeting sequence for the first time, cyclic structure based on RGD sequence both improves stability and cell penetrability. In addition, cyclic RGD peptide with two disulfide bonds possesses stronger vascular-targeting ability than those with one.

Non-basic amino acid substitution is another modification strategy widely applied in peptide design. D-amino acid substitution is commonly used to protect peptides from degradation though it may compromise the bioactivity of the original L-peptide [119]. Besides, β- and γ-amino acids can be also inserted in the sequence, forming complex secondary structures to provide greater thermal and enzymatic stability [120]. Recently, a macrocyclization was applied to an all-D linear α-helical peptide by introducing a hydrocarbon staple and improved target binding ability, proteolytic stability and increased cellular activity were demonstrated [121].

Bicycle peptide is a promising new class of molecules for targeted delivery of payloads into tumors, which is typically between 9 and 20 amino acids long and has 3 cysteine residues within the sequence. These cysteine residues react with a small molecule linker to constrain the peptide in a rigid conformation to construct bicycle toxin conjugates (BTCs) [122]. These conjugates exhibit several advantages over ADCs including deeper tumor penetration, rapid extravasation and slower renal clearance. Several BTCs are in clinical trials including BT1718, BT5528 and BT8009 [123, 124]. Especially, BT7480, a novel, first-in-class, Nectin-4/CD137 *Bicycle* tumor-targeted immune cell agonist™ (*Bicycle* TICA™) was recently developed [125].

Current tumor-targeting peptides come from two sources: natural plants or animals, and chemical synthesis or peptide libraries from phage display technologies and other screening technologies. A phage display peptide library is a powerful tool for the discovery of specific ligands with high receptor affinity. However, one disadvantage is that the technology produces peptides with a predetermined length and only from natural amino acids [126].

A synthetic peptide library is another method to obtain the tumor-targeting peptide, and the one-bead-one-compound (OBOC) method has made a particular impact. The OBOC method is based on a “mix and split” technique and enables the preparation of peptide libraries with 10^6^–10^8^ different peptides [127–129]. The synthetic flexibility of the OBOC method and the size of its libraries make it an ideal optimization tool for leading peptides previously discovered by phage display or other methods [127].

It should be pointed out that most of the existing peptides lack intrinsic activities on the signaling pathways unlike the paradigm of the antibody component of ADCs. As a complicated disease driven by multiple factors, PDCs based on the peptides with multiple functions will exert superior efficacy for tumor therapy, which will be discussed in the following section.

#### Optimization of payload and linker units

The cytotoxic payload is devoted to eliciting cell killing of the targeted tumor cells or tissues. The first generations of ADCs using DOX as the payload resulted in low clinical activity. Due to biodistribution, uptake, and loss of conjugation in circulation, it is estimated that only 1–2% of ADC payload reach the intracellular target [130]. Thus, the potency of the payload must be high (ideally in the subnanomolar range) to eradicate the target cells even at a lower accumulated concentration. Recently, high potent PBD dimers are emerging for ADC design, enabling complete regressions to be achieved in multiple pre-clinical in vivo models following just a single intravenous administration of ADC [131–134]. This increased potency also provides the ability to target low-copy number antigens, which may be particularly important for the treatment of solid tumors. Loncastuximab tesirine targeting CD19 for the treatment of B cell lymphomas has been approved in 2021 [135]. However, vadastuximab talirine targeting CD33 and rovalpituzumab tesirine targeting DLL3 were discontinued following pivotal studies. Considering that true tumor-specific antigens are rare, clinical outcomes of ADC may depend not only on the level of expression of the target antigen on normal cells but also on its relative functional importance on key organs, especially when using a highly potent warhead, such as PBD dimers [136]. Concern over the potency of the PBD dimers has also led to the suggestion that lower potency drugs may be required, particularly when there is a significant level of antigen expression on critical normal cells, such as HER2 in pulmonary tissue. Trastuzumab deruxtecan targeting a topoisomerase1 inhibitor with a drug-to-antibody ratio (DAR) of 8 gained accelerated approval in December 2019, the superior efficacy could be attributed to efficient tumor penetration and intracellular linker cleavage after ADC internalization [137, 138].

Recently, other types of payloads are emerging such as toxic proteins, cytokines, PROTACs and oligonucleotides, which can also be earned for the next generation of therapeutic PDCs [138].

Another crucial aspect of the drug conjugate design involves the linker, which should be carefully determined so as not to perturb the binding affinity of the peptide to its receptor and the drug’s efficacy. An inappropriate linker may impede the release of the drug from the PDC in the circulation and therefore diminish its overall therapeutic potency. Linkers utilized in PDCs include enzyme cleavable (ester, amide, and carbamate), acid cleavable (hydrazone and carbonate), reducible disulfide, and non-cleavable, which has been thoroughly reviewed for better PDC design [139].

### Innovating action mechanisms to cope with drug resistance

The development of drug resistance is a complicated and multifaceted process associated with enhanced efflux of drugs, elevated metabolism of xenobiotics, enhanced DNA repair capacity, signaling pathway compensation, target change, cell death inhibition and many more [140]. Drug resistance greatly contributes to chemotherapy failure in cancer therapy. As for ADCs, numerous efforts have been made to overcome the resistance. Since one of the most frequent mechanisms of resistance to ADCs is increased expression of drug efflux pumps and one strategy is to adopt the cytotoxic agent for drugs or toxins that are poor efflux substrates. Vadastuximab talirine, an anti-CD33 antibody coupled to PBD, showed robust activity in acute myelocytic leukemia (AML) animal models [131]. Trastuzumab deruxtecan, using a novel DNA topoisomerase I inhibitor, can overcome T-DM1 resistance caused by aberrant expression of ATP-binding cassette (ABC) transporters in HER2-positive gastric cancer [141]. Another strategy involved the linker modification to increase the hydrophilicity, based on the fact that MDR1 transports hydrophobic compounds more efficiently than hydrophilic compounds. Sulfo-SPDB [142] and mal-PEG4-N-hydroxysuccinimide are examples of polar linkers that have shown improved potency against MDR1^+^ models [143]. 

New formats of mAbs like bispecific / biparatopic ADCs have been developed to overcome resistance. The first biparatopic ADC, targeting two nonoverlapping epitopes on HER2 induced HER2 receptor clustering, which in turn promoted robust internalization and degradation, and also demonstrated antitumor activity in T-DM1-resistant tumor models [144]. Moreover, a bispecific antibody that binds HER2 and the prolactin receptor at the cell surface dramatically enhanced the cell-killing activity of a noncompeting HER2 ADC [145].

The afore-mentioned options in coping with ADCs resistance are worth learning for future PDCs construction. Some beneficial attempts have been made to improve the efficacy of PDCs. For example, LTP-1, a THP-CPP-PTX conjugate, by linking PTX with a multifunctional peptide consisting of a THP and a CPP, significantly enhanced the cytotoxicity and potentially counteracted PTX-resistance [146].

In addition to enhanced efflux of drugs, bypass compensation due to the abnormal activation of the downstream pathway or connecting signaling pathways likewise play an important role in mediating drug resistance. Notably, the more potent target inhibitors are used, the more frequently bypass tracks are likely to develop [147]. In addition to targeted delivery, more attention should be paid to how to deal with the compensatory alternations of intracellular signaling pathways under drug resistance.

It should be noted that most of the existing peptides whether screened from the phage display or synthetic peptide library lack intrinsic activities to influence the intracellular signal transduction, which is different from that of the antibody moieties employed in the ADCs construction. A targeting peptide that can affect multiple pathways simultaneously may greatly enhance the therapeutic efficacy.

We successfully identified a heptapeptide (P7) by phage display technique, which not only specifically binds to heat shock protein 90 (Hsp90) overexpressed on the cell surface [148], but also reduces the intracellular Hsp90 level in non-small cell lung cancer (NSCLC) cells [149]. Hsp90 is a molecular chaperone that maintains the structural and functional integrity of various client proteins involved in signaling and many other functions of cancer cells, making it a controller of signaling pathways. Hsp90 is also closely related to tumor treatment resistance. When tumor cells are exposed to treatments such as ionizing radiation or alkylation agents, the heat shock protein family is the first line of defense to maintain DNA integrity and cell integrity [150]. Various drug-resistant cancer cell lines increased expression of Hsp90 and concomitantly increased activations of pro-survival signaling pathways and cell cycle progression. The increased Hsp90 expression along with its client proteins, EGFR, IGF-1R, and Src, promotes autophagy in cancer cells and confers drug resistance [151]. In addition, Hsp90 regulates the expression of various drug-resistant genes, including LRP, GST-π, p53, bcl-2, survivin, ERCC1, XRCC1, BRCA1, and BRCA2 [152]. Numerous Hsp90 inhibitors have been investigated, however none have been approved by FDA due to side toxic effects.

**Fig. 4:**
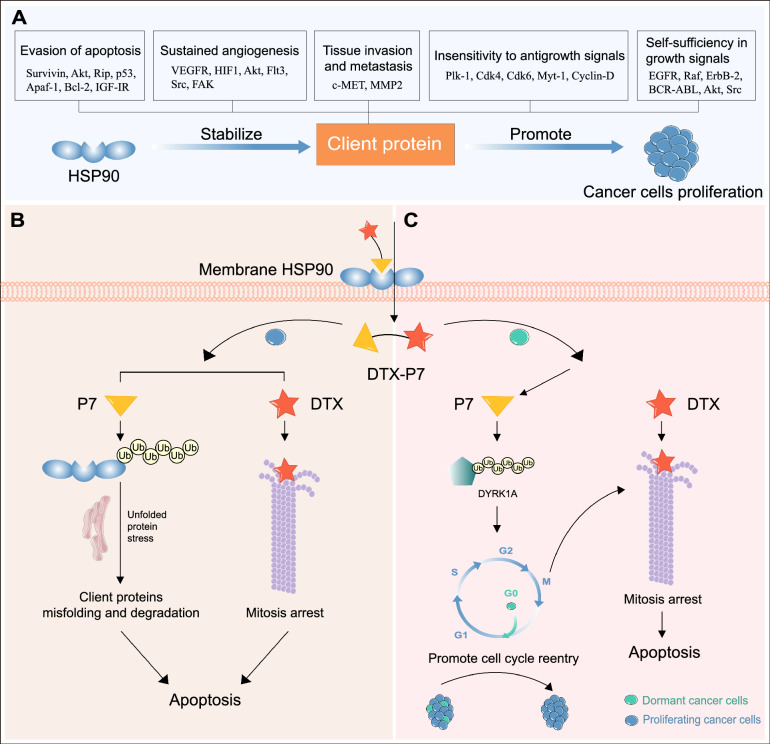
Dual functional peptide endows DTX-P7 with significant anti-tumor effects. **A** Hsp90 is involved in multiple cellular signaling pathways that regulate apoptosis and cell survival, making it an important therapeutic target by modulating the maturation and stability of about 400 client proteins. **B** DTX-P7 specifically binds to cell surface Hsp90. For rapidly proliferating cells, DTX-P7 accelerates the degradation of Hsp90 through lysosome- and proteasome-dependent pathways, which induces unfolded protein response and subsequently promotes apoptosis. DTX promotes tubulin assembly into microtubules and inhibit their depolymerization, thus blocking cells in G2/M phase. The synergistic effect can be observed during the treatment of DTX-P7. **C** For dormant cells, DTX-P7 suppresses survival of quiescent/slowly proliferating cells via degradation of DYRK1A and subsequent cell cycle re-entry

Recent studies demonstrate that Hsp90α is also expressed on the tumor cell surface [153–157], making it feasible to construct a peptide-conjugate to realize multiple anti-tumor effects. These obtained results spurred us to construct a novel peptide drug conjugate (DTX-P7) by conjugating docetaxel (DTX) with P7. We demonstrated that DTX-P7 preferentially suppressed tumor growth compared with DTX in vivo. Meanwhile, the pharmacokinetic analysis showed that DTX-P7 exhibited a favorable distribution to tumor tissues and a long circulation half-life. Furthermore, we revealed a distinctive mechanism whereby DTX-P7 induced unfolded protein response and eventually promoted apoptosis, leading to cell death. More importantly, we found that DTX-P7 promoted the cell cycle re-entry of low-proliferative cancer stem cells (CSCs) and subsequently killed them, exhibiting a “proliferate to kill” pattern (Fig. 4B, C) [149]. CSCs are a notoriously quiescent subpopulation of cells within heterogeneous tumors exhibiting self-renewal, differentiation and drug-resistant capabilities leading to tumor relapse [158].

It is completely possible that there are other controllers of multi-signaling pathways similar to Hsp90. The PDC constructed based on the peptide, not merely targeting but also affecting intracellular multi-signaling pathway transduction, will provide superior selectivity and higher efficacy.

## Conclusion and future perspectives

With the growing understanding of cancer cell biology, the clinical efficacy has been improved from the single-drug treatment of traditional chemotherapy drugs to drug combinations, targeted therapy, and conjugates treatment. Although the approval of ADCs realizes great improvement in cancer therapy, we should be soberly aware there are still some limitations or challenges in the ADCs’ development. PDCs are small in size, easily synthesized as single homogeneous entities that are well-characterized for precise large-scale production, and can provide improved pharmacokinetic profiles. The progress in PDCs is still in the early stage, but with promising potential. A systemic rethink of pitfalls in ADCs’ design is beneficial for future PDCs’ construction. It is particularly vital to identify the specific neoantigen and emphasize the intracellular signal alteration for designing potent engineered conjugates and biological entities to boost efficient therapies for cancer treatment.

## Data Availability

Not applicable.

## Abbreviations

ABC: ATP-binding cassette
ADC: Antibody–drug conjugate
AML: Acute myelocytic leukemia
BNCT: Boron neutron capture therapy
BTC: Bicycle toxin conjugate
CLR: C-type lectin receptor
CPP: Cell-penetrating peptide
CPT: Camptothecin
CSC: Cancer stem cell
DAR: Drug-to-antibody ratio
DDS: Drug delivery system
DOX: Doxorubicin
DTX: Docetaxel
EC_50_: Concentration for 50% of maximal effect
ECM: Extracellular matrix
EGFR: Epidermal growth factor receptor
Fc: Fragment crystallizable
FcRn: Neonatal Fc receptor
FcγR: Fc gamma receptor
GEM: Gemcitabine
GnRH: Gonadotropin-releasing hormone
GnRH-R: Gonadotropin-releasing hormone receptor
HER: Human epidermal growth factor receptor
HSP: Heat shock protein
IC_50_: Half maximal inhibitory concentration
LHRH: Luteinizing hormone-releasing hormone
mAb: Monoclonal antibody
mCRPC: Metastatic castration-resistant prostate cancer
MDR: Multi-drug resistance
miRNA: Micro RNA
ML: Machine learning
NSCLC: Non-small cell lung cancer
OBOC: One-bead-one-compound
PBD: Pyrrolobenzodiazepine
PDC: Peptide-drug conjugate
PGS: Prodigiosin
PRRT: Peptide receptor radionuclide therapy
PTX: Paclitaxel
RDC: Radionuclide-drug conjugate
SSTR: Somatostatin receptor
TAA: Tumor-associated antigen
TF: Transcription factor
THP: Tumor-homing peptide
TNBC: Triple-negative breast cancer
TSA: Tumor-specific antigen
